# Retraction: Impacts of rock properties on Danxia landform formation based on lithological experiments at Kongtongshan National Geopark, northwest China

**DOI:** 10.1371/journal.pone.0223286

**Published:** 2019-09-26

**Authors:** Haibin Zhu, Hua Peng, Zhixin Pan, and the PLOS ONE Editors

Following publication of this article [1], the corresponding author requested its retraction owing to the following issues:

- The map shown in Fig 2 was modified from [2] without permission, and the source of the image was not cited;
- The map shown in Fig 3 was modified from [3] without permission, and the source of the image was not cited;
- The photographs in Fig 4c and 4d were provided by the leader of the Administrative Committee of Kongtongshan and reproduced with permission from the copyright holder, but permission was not obtained in written form and the source of the images was not stated;
- Information reported in the “Geologic setting” section in [1] was previously described in section 2.3 of [3], and the source of this information was not cited;
- The reported X-ray diffraction (XRD) results in Fig 6 were not obtained from experiments carried out by the authors at Sun Yat-sen University as reported. The data, previously published in Figure 5.4 of [3], were provided to the corresponding author and used without permission and without citation;
- The reported scanning electron microscopy (SEM) photographs in Fig 7 were not obtained from experiments carried out by the authors at Sun Yat-sen University as reported. The images, previously published in Figure 5.8 of [3], were provided to the corresponding author and used without permission and without citation;
- The reported inductively coupled plasma mass spectrometry (ICP-MS) analysis in Table 3 was not carried out by the authors at Sun Yat-sen University as reported. The reported data are from [3] and were used without permission and without citation.

The corresponding author has indicated that XRD and SEM experiments were carried out by the authors at Sun Yat-sen University, but that the images and data previously published in [3] were used in place of the data that were collected by the authors.

The corresponding author apologizes for misusing data from others’ research and failing to follow academic standards in publication ethics.

In view of the misrepresentation of the results and data reported in [1], the *PLOS ONE* Editors and the authors retract this article.

Figs 2, 3, 4c, 4d, 6, and 7 report material which are not offered under a CC-BY license and are therefore excluded from this article’s [1] license. At the time of retraction, the article [1] was republished to note this exclusion in the legends of Figs 2, 3, 4, 6, and 7, and in the article’s copyright statement.

HZ, ZP agreed with the retraction. HP is deceased.
